# The Modulation of Working-Memory Performance Using Gamma-Electroacupuncture and Theta-Electroacupuncture in Healthy Adults

**DOI:** 10.1155/2021/2062718

**Published:** 2021-11-16

**Authors:** Meng Ren, Jingjing Xu, Jingjun Zhao, Sicong Zhang, Wenjing Wang, Shutian Xu, Zhiqing Zhou, Xixi Chen, Songmei Chen, Yuanli Li, Chunlei Shan

**Affiliations:** ^1^Yueyang Hospital of Integrated Traditional Chinese and Western Medicine, Shanghai University of Traditional Chinese Medicine, No. 110, Gan-He Road, Shanghai, China; ^2^School of Rehabilitation Medicine, Shanghai University of Traditional Chinese Medicine, No. 1200, Cai-Lun Road, Shanghai, China; ^3^Guangzhou Xuguan Clinic of Traditional Chinese Medicine, No. 865, Zhonghua Road, Guangzhou, China; ^4^Guangzhou Xinhua College, No. 19, Longdonghuamei Road, Guangzhou, China; ^5^Engineering Research Center of Traditional Chinese Medicine Intelligent Rehabilitation, Ministry of Education, No. 1200, Cai-Lun Road, Shanghai, China; ^6^Shanghai No. 3 Rehabilitation Hospital, No. 100, Jiao-Cheng, Shanghai, China

## Abstract

Working memory (WM), a central component of general cognition, plays an essential role in human beings' daily lives. WM impairments often occur in psychiatric, neurodegenerative, and neurodevelopmental disorders, mainly presenting as loss of high-load WM. In previous research, electroacupuncture (EA) has been shown to be an effective treatment for cognitive impairments. Frequency parameters are an important factor in therapeutic results, but the optimal frequency parameters of EA have not yet been identified. In this study, we chose theta-EA (*θ*-EA; 6 Hz) and gamma-EA (*γ*-EA; 40 Hz), corresponding to the transcranial alternating-current stimulation (tACS) frequency parameters at the Baihui (DU20) and Shenting (DU24) acupoints, in order to compare the effects of different EA frequencies on WM. We evaluated WM performance using visual 1-back, 2-back, and 3-back WM tasks involving digits. Each participant (*N* = 30) attended three different sessions in accordance with a within-subject crossover design. We performed *θ*-EA, *γ*-EA, and sham-EA in a counterbalanced order, conducting the WM task both before and after intervention. The results showed that d-prime (d′) under all three stimulation conditions had no significance in the 1-back and 2-back tasks. However, in the 3-back task, there was a significant improvement in d′ after intervention compared to d′ before intervention under *θ*-EA (F [1, 29] = 22.64; *P* < 0.001), while we saw no significant difference in the *γ*-EA and sham-EA groups. Reaction times for hits (RT-hit) under all three stimulation conditions showed decreasing trends in 1-, 2-, and 3-back tasks but without statistically significant differences. These findings suggest that the application of *θ*-EA might facilitate high-load WM performance.

## 1. Introduction

Working memory (WM) can be defined as the ability to maintain and manipulate new and stored memory information for a short period of time [1]. WM impairment often occurs in many mental and neurodegenerative disorders, including depression, schizophrenia, Alzheimer's, and Parkinson's diseases, and some neurodevelopmental disorders such as attention deficit hyperactivity disorder and autistic spectrum disorder [2–6]. Working-memory load (WM load) is the amount of information that must be held in the mind at any given time [7]. Many previous studies have shown that WM is especially impaired when memory load is high [5, 8–10]. The n-back task, a typical WM paradigm, is widely used to manipulate WM processing load. Compared with young adults, older adults generally exhibit poor WM performance in the high-load n-back task [11]. Several studies have found that people with WM impairment also show abnormal cortical electrical activity [12, 13].

The electrical activity of neurons and neuron clusters is the basis of cortical excitability and is strongly associated with cognitive activities [14]. There is considerable evidence for an association between increased cortical oscillations at the gamma (*γ*; >30 Hz) and theta (*θ*; 3–8 Hz) frequencies and increased WM load [15–22]. Neurophysiological studies suggest that WM is driven by *θ*-frequency band oscillations in frontal-parietal networks, while *θ*-oscillations increase with added WM load [23–25]. A previous study showed that *θ*-power might be critical for the encoding of new information, especially reflecting cognitive-control functions in WM [26]. It has also been found that frontal *θ*-oscillations play a causal role in prioritizing WM representations and that the power of frontal *θ* increases during active maintenance of WM information until the information is retrieved [27]. Oscillations in the gamma band also play an important role in maintaining WM information. Considerable evidence shows that cortical and hippocampal *γ*-power increases with a greater number of letters, digits, or faces to remember [7, 28, 29]. Elevated *γ*-power is associated with the maintenance of multiple items in WM, indicating a role for *γ* in WM as well as in perception. Additionally, previous research has shown that cortical oscillatory neural activity in the *γ*-frequency range was related to attention control and that successful attentional processing depends on the *γ*-phase [30–34]. Attention control is also an essential component of WM, especially for high-load WM tasks, and participates in the whole process of WM [10]. In conclusion, the *θ*-band and *γ*-band are crucial to WM, and, therefore, regulating electrical activity at these frequencies could enhance WM.

In this study, we chose electroacupuncture (EA) as an intervention for regulating electrical activity in the cerebral cortex. EA is a treatment method based on traditional acupuncture technology, delivering a specific current to acupoints. It has many advantages, such as low cost, few side effects, and high safety, and it has been widely used in clinical practice. One meta-analysis showed that EA can effectively improve cognitive function [35]. Another study evaluated the effect of EA delivered to the Baihui (DU20) and Shenting (DU24) acupoints at a frequency of 80 Hz on vascular cognitive impairment with no dementia (VCIND); the results showed a clear improvement in patients' cognitive ability and quality of life [36]. In another study, EA was applied to the Shenting (DU24), Baihui (DU20), Sishencong (EX-HN1), and Fengchi (GB20) acupoints in mildly cognitively impaired (MCI) patients once every other day for 8 weeks; the EA improved patients' short-term memory abilities more significantly than a Nimodipine treatment regimen [37]. Although many studies have found that EA can improve cognitive function, some previous studies have shown that EA frequencies and acupoints were not consistent among them, as the choice of EA parameters often depends on the experience of the investigators.

In this study, we delivered *θ*-EA and *γ*-EA to the Baihui (DU20) and Shenting (DU24) acupoints in order to assess the effect of EA on WM. Neuroscience research has found that neural oscillations play an important role in cognitive function. In previous studies of EA, the frequency selected did not consider the characteristics of the electrical activity of the cortex but was simply divided into high and low frequencies. Therefore, we determined EA frequencies by referring to the theoretical logic of the frequencies of transcranial alternating-current stimulation (tACS). Previous evidence has suggested that applying tACS in the *θ*-frequency and *γ*-frequency bands can have a performance-enhancing effect on WM, including reaction times (RT) and accuracy [38–40]; we therefore applied EA at 6 and 40 Hz, the respective frequencies of these two bands. The choice of acupoints is also crucial; thus, we chose DU20 and DU24, which are located above the frontal and parietal lobes of the brain. Both are points in the Governor Vessel; they are the main points for regulating brain function and can be stimulated to treat many psychiatric and neurological diseases. Based on Chinese acupuncture theory, acupuncture at DU20 and DU24 can regulate the qi of the Governor Vessel, clear the mind, lift the spirits, and nourish yang [41]. Other studies have found that EA at DU20 and DU24 can directly regulate the functional activities of the frontal lobe [42–44], thereby improving the patient's cognitive function. EA at DU20 and DU24 appears to have significant benefits on memory and moderate benefits on executive functions and problem-solving in patients with schizophrenia [45]. Another study showed that stimulating the DU20 and DU24 acupoints can cause a nerve conduction effect and ameliorate cognitive impairment [46]. Collectively, these studies provided the basis for our choice of these two acupoints.

In the current study, we investigated changes in WM performance after *γ*-EA and *θ*-EA using n-back tasks, with a sham-EA group being used as the control group. We hypothesized that *θ*-EA could improve the accuracy and *γ*-EA could reduce the RT.

## 2. Methods

### 2.1. Participants

We recruited 30 healthy participants (15 males and 15 females) aged 18–24 years (mean (M) ± standard deviation (SD), 21.5 ± 1.8 years) in this study. All of the participants were right-handed, had never received EA before, and had no reported neurological or psychiatric disorders, drug abuse problems, or current pregnancies. All of the participants underwent the Montreal Cognitive Assessment (MoCA) to ascertain a lack of cognitive impairment (MoCA ≥26) [47]. The participants were informed about all aspects of the study, and written informed consent was obtained from all the participants before the start of the study.

Prospective participants who met any of the following criteria were excluded: (a) local skin infections, ulcers, or scars at the selected points or being overly sensitive to acupuncture; (b) severe bleeding disorders and coagulation disorders; (c) history of epilepsy; (d) diseases or medical history that may affect cognitive function; (e) severe vision or hearing impairment; (f) refusal to cooperate with the assessment; (g) people with a history of smoking or alcohol abuse.

Ethical approval was granted by Yueyang Hospital of Integrated Traditional Chinese and Western Medicine, Shanghai, China (Ethics No. 2019–121). Prior to the start of this research, it was registered with the Chinese Clinical Trial Registry (https://www.chictr.org.cn/index.aspx) on 8 December 2019, ChiCRT1900028025.

### 2.2. Procedure

**Figure 1:**
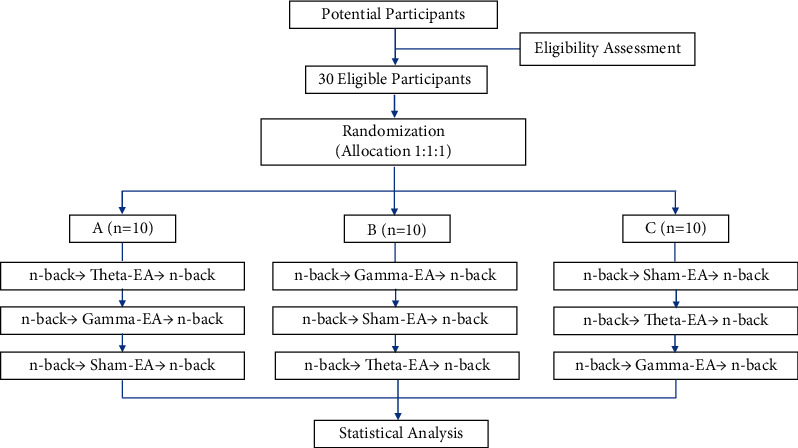
Each participant underwent three experimental sessions spaced at least 7 d apart; the session order was randomized. *γ*-EA, *θ*-EA, and sham-EA were applied for 30 min. Working-memory assessments before and after stimulation consisted of the 1-, 2-, and 3-back tasks.

This was a randomized, repeated-measure, single-blinded study with a within-subject crossover design. Gender was balanced between groups, and the sequence of stimulation conditions was randomized and counterbalanced across participants. The order of n-back tasks was also balanced in the different groups, and the order in the post-EA block was consistent with that in the pre-EA block. We conducted sessions 7 days apart to avoid the possibility of carryover effects of stimulation [48]. Each session involved the delivery of *γ*-EA, *θ*-EA, or sham-EA for 30 min. WM performance was assessed using 1-, 2-, and 3-back tasks both prior to and following stimulation (Figure 1). All sessions were conducted during the day. A qualiﬁed acupuncturist performed all of the interventions. In this study, we assessed the participants' feelings during the intervention period verbally to ensure that the blinding procedure was successful. We found that the participants could not distinguish between the three stimulation conditions, suggesting that the blinding procedure was reliable.

### 2.3. Electroacupuncture

**Figure 2:**
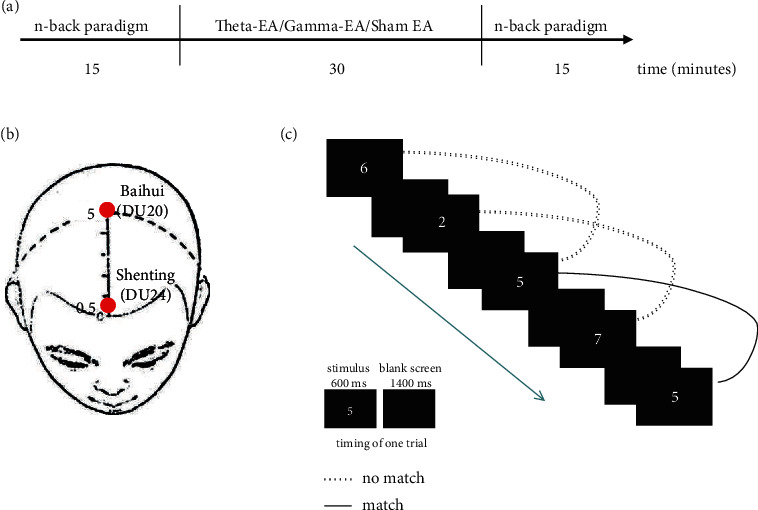
(a) Procedure during one session; (b) acupuncture points used; (c) two-back WM task.

Participants in the EA group received acupuncture based on traditional Chinese medicine meridian theory at (1) the Baihui (DU20) acupoint, located at the intersection of the line connecting the apexes of the two auricles and the median line of the head, 7 cun (1 cun = 3.33 cm) directly above the posterior hairline, and 5 cun behind the anterior hairline, and (2) the Shenting (DU24) acupoint, located 0.5 cun directly above the midpoint of the anterior hairline (Figure 2(b)). The insertion angle was about 10°–20° (between the needle and the scalp), and the insertion depth of the disposable needle (0.35 mm × 40 mm, Hwato, Suzhou, China) was about 0.3–0.5 cun. After insertion, the acupuncturist gently twisted the needle for 1 min. After “deqi” (a feeling of soreness, tingling, or bloating) was achieved, the needle at each point was then connected to an SDZ-III EA device (Hwato, Suzhou, China) to provide electrical stimulation. The device was placed behind the patient to ensure that the patient did not know the specific frequency parameters of the intervention. An experienced acupuncturist with a Medical Practitioner's Qualification Certificate performed all acupuncture in this study. The participants did not receive any other interventions in addition to needling [49].

### 2.4. Gamma-EA

EA was applied for 30 min with a continuous current at a frequency of 40 Hz. Current intensity was based on the subject's tolerance, preferably with the skin around the acupoints shivering mildly without pain. This treatment was only performed once to explore its immediate effects.

### 2.5. Theta-EA

EA was applied for 30 min with a continuous current at a frequency of 6 Hz. Other parameters were the same as those for the *γ*-EA group. This treatment was only performed once to explore its immediate effects.

### 2.6. Sham-EA

Acupuncture points and operational procedures of the sham-EA group were consistent with those of the EA groups. The only difference was that the electrode connected to the needle body was in a no-current state, using a special wire that could not be powered, although its appearance was the same as that of ordinary wires [50]. This treatment was only performed once to explore its immediate effects.

### 2.7. Working-Memory Task: N-Back

N-back is the classic paradigm of WM processing load. During each session, we asked the participants to perform a visual n-back task, involving digits, twice for 15 min (5 min of 1-back, 5 min of 2-back, and 5 min of 3-back task). In each test, when the current digit was the same as that presented one, two, or three trials earlier (depending on the task), the participants were asked to respond by pressing a button (the *j* button for the same number and the *f* button for a different number) as quickly as possible. Each test consisted of 200 trials and included 40 target stimuli. Each stimulus was presented for 600 ms, followed by an empty screen for 1400 ms as the stimulus interval. The task was performed using E-Prime software version 3.0 (Psychology Software Tools [PST] Inc., Pittsburgh, PA, USA). Digits were 2 cm tall and white in color; they were presented against a black background on a 24-inch screen. The participants were seated at a distance of 0.8 m from the screen, resulting in a visual angle of 1.001°.

### 2.8. Statistical Analysis

In this study, we analyzed reaction times for hits (RT-hit), the most commonly reported method of evaluating performance on n-back tasks [51]. We also calculated d-prime (d′) for each n-back digital task. D′ is a well-established measure for quantifying WM performance and determining whether it has changed over time. Based on signal detection theory, d′ is a net score that takes into account the range for both components by calculating the relative proportion of hits minus false alarms, thus providing a dependable measure of the participant's ability to distinguish between items [52]. Correct responses were regarded as hits. False alarms occurred when the participant erroneously responded that the current digit had appeared before.

Prestimulation performance for all of the sessions was quantified using RT-hit and d′. We compared the performance between stimulation types via one-way analysis of variance (ANOVA), using the within-subject factor of stimulation type (*θ*-EA, *γ*-EA, and sham-EA). There was no significant difference between the various stimulation types before intervention (1-back RT-hit: *F* [2, 87] = 0.641, *P*=0.529, d′: *F* [2, 87] = 0.024, *P*=0.977; 2-back RT-hit: *F*[2, 87] = 0.145, *P*=0.865, d′: *F* [2, 87] = 0.21, *P*=0.811; 3-back RT-hit: *F* [2, 87] = 0.127, *P*=0.881, d′: *F* [2, 87] = 0.62, *P*=0.54).

Initially, three-factor repeated-measure ANOVA analyses including the factors WM load (1-back, 2-back, and 3-back), time (before and after), and stimulation type (*θ*-EA, *γ*-EA, and sham-EA) were conducted separately for RT-hit and accuracy (d′). According to this initial analysis, there were no significant interactions in WM load ^*∗*^ time ^*∗*^ stimulation type (RT-hit: *F* [4, 116] = 0.719, *P*=0.581; d′: *F*[4, 116] = 1.108, *P*=0.356). Thus, we performed a secondary analysis.

We performed a two-way repeated-measure ANOVA for RT-hit, using the within-subject factors of stimulation type (*θ*-EA versus *γ*-EA versus sham-EA) and assessment time (before simulation versus after stimulation; *N* = 30; within-subject crossover design) for the 1-back, 2-back, and 3-back tasks separately. Mauchly's test of sphericity was used to assess the assumption of sphericity; when sphericity was rejected, we implemented the Greenhouse–Geisser correction. These ANOVAs were also calculated for accuracy (d′). We used SPSS version 24 (IBM Corp., Armonk, NY, USA) and in-house MATLAB (version R2013b) scripts (MathWorks, Natick, MA, USA) to perform statistical analysis. Only the behavioral data of this study were analyzed in this manuscript.

## 3. Results

### 3.1. Reaction Time for Hits

**Figure 3:**
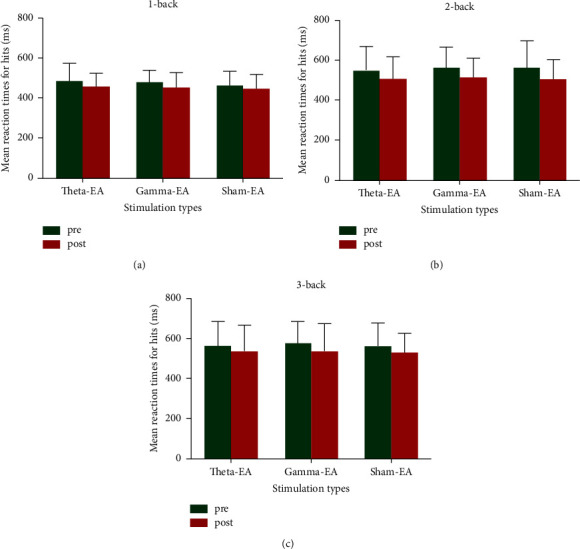
Mean RT-hit measured before and after stimulation for each stimulation type: (a) 1-back task; (b) 2-back task; (c) 3-back task.

Two-way repeated-measure ANOVA did not reveal a significant interaction between stimulation type and assessment time (1-back: *F* [2, 58] = 0.56, *P*=0.57; 2-back: *F* [2, 58] = 0.38, *P*=0.69; 3-back: *F* [2,48.34] = 0.21, *P*=0.77, Greenhouse–Geisser corrected: *ε* = 0.83; Figure 3). However, the simple main effect of assessment time was significant (1-back: *F* [1, 29] = 16.39, *P* < 0.001; 2-back: *F* [1, 29] = 39.28, *P* < 0.001; 3-back: *F* [1, 29] = 13.68, *P*=0.001), indicating a significant effect of practice, independent of stimulation type. The results are shown in Table 1.

### 3.2. d-Prime

**Figure 4:**
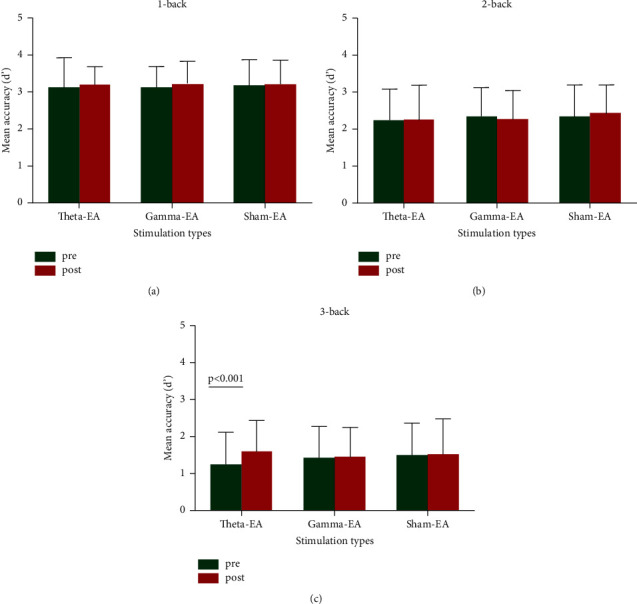
Mean accuracy reflected in d′ values measured before and after stimulation for each stimulation type: (a) 1-back task; (b) 2-back task; (c) 3-back task.

We analyzed changes in d′ via a method similar to that used for RT-hit. Two-way repeated-measure ANOVA showed no interaction of time and stimulation type in the 1-back task (*F* [2, 58] = 0.77, *P*=0.93; Figure 4(a)) or 2-back task (*F* [2, 58] = 0.60, *P*=0.55; Figure 4(b)) and no main effect of stimulation group or time. There was an interaction of time and stimulation type in the 3-back task (*F* [2, 58] = 3.55, *P* = 0.035), and a simple-effect test for time showed significant improvement only in the *θ*-EA group (*F* [1, 29] = 22.64, corrected *P* < 0.001; Figure 4(c)). The results are shown in Table 2. Bonferroni correction was performed for multiple comparisons.

## 4. Discussion

Despite the wide use of EA to improve cognitive function, there is still a lack of clarity in the choice of stimulation frequency in previous studies. The study of EA based on neural oscillation frequencies specific to WM has not been reported before. We assumed that a specific frequency of EA might cause a specific frequency of neural oscillation. Therefore, in the current study, we chose the *θ*-EA and *γ*-EA frequencies based on the characteristics of cortical electrical activity to modulate the WM of healthy subjects. We also observed whether EA had different effects on n-back tasks with different loads. We estimated the impact of EA on WM using a visual n-back test that involved digits. Our findings are summarized as follows.

RT-hit represented the time taken by the subject to respond correctly after the stimulus appeared. Previous studies have suggested that RT-hit values in n-back tasks are a representation of the subject's attention; the lower the value of RT-hit, the higher the subject's alertness [51]. Many reports have shown that *γ*-oscillations are related to perception and attention control [31, 53]. Successful allocation of attention is characterized by elevated *γ*-activity [31]. Therefore, we expected that *γ*-EA could improve RT-hit in n-back tasks. The results of the current study were inconsistent with this expectation, especially that of EA at *γ*-frequency. There could be many reasons for these results. First, they are similar to those of an earlier study [51]. Although the authors of that study chose transcranial electrical stimulation (TES), a popular intervention method, they did not find that TES improved RT-hit under any of the three stimulation types used (tACS, transcranial direct-current stimulation [tDCS], and sham stimulation). They further analyzed the data and found that there might have been an improvement in performance due to practice, independent of stimulation type. Although in this study we familiarized subjects with the test procedure before the start of the formal experiment, the practice effect was not avoided and therefore might also have led to the results that we obtained. Another study found a WM load-related increase in the *γ*-band that was localized to the right intraparietal lobule and left Brodmann area 9 (BA9) [20]. However, the locations of DU20 and DU24 are different, and different stimulation sites result in different effects. Moreover, we enrolled a young, healthy participant group in this study, as our aim was to compare the direct effects of our methods without confounding factors. Our participants could be seen as high-performing as all of them were college students, potentially explaining the small improvement in RT-hit with stimulation. Therefore, the existence of a “ceiling effect” is reasonable in the 1- and 2-back tasks, which would prevent a subsequent improvement in RT-hit. Conversely, the 3-back task is a high-load task in which the participants might focus on accuracy rather than the speed of response, meaning that RT-hit times in the 3-back task were longer and might have not differed significantly among the three groups before and after intervention.

We also observed differences in accuracy among the three intervention programs in terms of the n-back task in this study. d′ is a well-established measure for reflecting the ability to differentiate between items; a higher d′ indicates that the signal/target is more easily detected and that the subject performs better. The offline effect of *θ*-EA stimulation (accuracy before versus after stimulation) showed a significant improvement in 3-back task performance, which we did not observe for the *γ*-EA and sham-EA stimulatory conditions. This outcome was partially in line with our expectations. An increase of *θ*-frequency band power can be consistently observed during WM maintenance. This increase is typically proportional to the amount of information being retained in the mind, with higher loads and/or effort resulting in higher *θ*-band power [19, 54–56]. For example, Gevins et al. [17] used an n-back task that allowed them to examine changes in activity as a function of the amount of information retained in the WM. They found that frontal-midline theta (FMT) power directly increased as WM load increased. Jensen and Tesche [57] conducted a magnetoencephalography (MEG) study of the Sternberg WM paradigm using digits as stimuli and found that the amplitude of *θ*-power increased as WM load increased. Alternatively, the n-back task might mainly depend on WM processing and cognitive control. A previous review suggests that the *θ*-frequency band might especially reflect cognitive-control functions in WM [26]. The *θ*-frequency range power has also been shown to be related to WM processing in n-back tasks [15]. Therefore, we expected that *θ*-EA could improve the accuracy of high-load tasks.

A few studies, including some on *θ*-TES modulating the accuracy of n-back, have also found that *θ*-tACS can improve the accuracy of WM [58, 59]. However, this influence was dependent on the number of n-back steps and the placement of the target electrode. When this electrode was placed over the left parietal site, the highest performance increases were observed in 2-back tasks; when it was placed over the left frontal area, a moderate performance increase in the 1-back task could be observed. No improvement in performance was found on the 3-back test [60]. The acupoints in our study were DU20 and DU24; based on modern anatomical findings, these two acupoints are in the projection area of the frontal midline (FM). In WM tasks, FMT power incrementation is a well-known phenomenon [17, 61–63]. One study using a complementary learning systems model [64] found increased FMT power during the execution of an n-back task. FMT oscillations were particularly correlated with the prefrontal and anterior cingulate cortices (PFCs and ACCs). The ACC is a key structure implicated in WM.

We found a significant improvement in accuracy in the 3-back task but not in the 1- or 2-back task. There are several potential explanations for this improvement, compared with those in other reports. First, notably, as the 1-back task is a low-load WM task in which accuracy peaks before intervention, it might therefore be difficult to capture the effects of intervention. The 2-back load task is also relatively simple for college students and thus may not be much more sensitive in terms of accuracy. Additionally, in previous WM studies, *θ*-frequency range activity was reported to be linked to WM load and task difficulty [57, 65–67]. Some studies also found a linear increase of FMT power with load only in the high-WM capacity group [68, 69].

We also found that there was an improving trend of RT-hit and accuracy in sham-EA in the study, which is in line with our expectation. The acupoints in our study were DU20 and DU24, which are closely related to advanced thinking, spirit, and memory. Stimulating the DU20 and DU24 acupoints can cause a nerve conduction effect and improve cognitive impairment. Lee et al. reported that acupuncture stimulation at the DU20 can regulate the expression of brain-derived neurotrophic factor (BDNF) and cAMP-response element-binding protein (CREB) in the hippocampus of rats to improve cognitive function [70]. BDNF has a significant impact on memory performance and altered function in WM circuitry. It has also been found that manual acupuncture can increase the content of acetylcholine in the brain and, at the same time, adjust monoamine neurotransmitters and expression levels of 5-hydroxytryptamine, dopamine, and acetylcholine esterase and improve the memory performance of model rats [71]. These may be the reasons for the effect of the sham-EA on WM in this study.

The findings of our study can facilitate further investigation into *θ*-EA, which is a potential means of enhancing WM performance. In future studies, we will also measure the effect of *θ*-EA and *γ*-EA on the physiological activity of the cortex via electroencephalogram (EEG) or functional magnetic-resonance imaging (fMRI). Existing evidence supports the notion that *θ*-tACS can significantly affect *θ*-amplitude, but no significant effect of *γ*-tACS on EEG amplitude has been found in any observed frequency band [72]. However, the relevant effects on event-related potentials (ERPs) and responsive brain areas are still unknown and need further research. The cumulative effect, that is, the effect of *θ*-EA and *γ*-EA, on WM behavioral activity after continuous intervention for several days or months also needs to be explored to determine the best intervention parameters for patients with brain function disorders.

## 5. Limitations

There are also some limitations in this study. First, moderate intensity was applied for both theta- and gamma-EA in this study. To improve precision, further research should make use of other new EA devices that can display the exact intensity number, such as the HANS-2000 device. Second, the participants were not asked to perform any task during EA, which might be related to the state-dependent effects of EA. We plan to compare the effect between performing tasks during EA simultaneously (“online”) and performing tasks after EA separately (“offline”) in the future. Third, although we used a tape measure to locate acupuncture points before each intervention, minor measurement errors may still exist. The participants received the three EA interventions on three different days, introducing multiple possibilities for measurement error. Further studies should ensure that the electrode placement is fully consistent in follow-up studies. Finally, the research subjects were limited to normal young people in this study; as a result, we are unsure whether similar effects would be observed in subjects with cognitive impairment. Additional clinical study of subjects with cognitive impairment should be done in the future.

## Figures and Tables

**Table 1:** Means and standard deviations (in parentheses) of reaction time for each n-back task, EA-condition, and for pre- and post-EA (*N* = 30).

	1-Back		2-Back		3-Back	
	Pre	Post	Pre	Post	Pre	Post
Theta-EA	485.80 (86.96)	454.87 (69.03)	550.28 (117.00)	506.76 (107.88)	562.84 (121.48)	536.79 (129.75)
Gamma-EA	476.97 (62.77)	454.08 (72.65)	562.04 (101.66)	513.16 (94.88)	575.60 (107.08)	537.27 (136.51)
Sham-EA	464.30 (69.96)	448.29 (67.78)	565.70 (127.45)	504.93 (97.23)	562.77 (111.12)	532.23 (93.64)
*F*	0.56		0.38		0.21	
*p*	0.57		0.69		0.77	

**Table 2:** Means and standard deviations (in parentheses) of *d*-prime for each n-back task, EA-condition, and for pre- and post-EA (*N* = 30).

	1-Back		2-Back		3-Back	
	Pre	Post	Pre	Post	Pre	Post
Theta-EA	3.14 (0.77)	3.20 (0.48)	2.24 (0.83)	2.25 (0.93)	1.26 (0.86)	1.59 (0.85)^*∗∗*^
Gamma-EA	3.12 (0.56)	3.24 (0.57)	2.36 (0.75)	2.27 (0.75)	1.43 (0.84)	1.44 (0.80)
Sham-EA	3.16 (0.71)	3.21 (0.65)	2.35 (0.82)	2.44 (0.74)	1.50 (0.86)	1.52 (0.95)
*F*	0.77		0.60		3.55	
*p*	0.93		0.55		0.035^*∗*^	

^∗^ *p* < 0.05; ^∗∗^*p* < 0.001.

## Data Availability

The data are available on http://www.chictr.org.cn/showproj.aspx?proj=46693.
